# Multi-Period Network Design Problem in Regional Hazardous Waste Management Systems

**DOI:** 10.3390/ijerph16112042

**Published:** 2019-06-08

**Authors:** Jun Zhao, Lixiang Huang

**Affiliations:** 1 School of Transportation and Logistics, Southwest Jiaotong University, Chengdu 611756, China; 2 School of Economics and Management, Chengdu Technological University, Chengdu 611730, China; amandahuang@163.com

**Keywords:** hazardous waste management, multi-period network design, multi-objective optimization, location, allocation

## Abstract

The management of hazardous wastes in regions is required to design a multi-echelon network with multiple facilities including recycling, treatment and disposal centers servicing the transportation, recycling, treatment and disposal procedures of hazardous wastes and waste residues. The multi-period network design problem within is to determine the location of waste facilities and allocation/transportation of wastes/residues in each period during the planning horizon, such that the total cost and total risk in the location and transportation procedures are minimized. With consideration of the life cycle capacity of disposal centers, we formulate the problem as a bi-objective mixed integer linear programming model in which a unified modeling strategy is designed to describe the closing of existing waste facilities and the opening of new waste facilities. By exploiting the characteristics of the proposed model, an augmented ε-constraint algorithm is developed to solve the model and find highly qualified representative non-dominated solutions. Finally, computational results of a realistic case demonstrate that our algorithm can identify obviously distinct and uniformly distributed representative non-dominated solutions within reasonable time, revealing the trade-off between the total cost and total risk objectives efficiently. Meanwhile, the multi-period network design optimization is superior to the single-period optimization in terms of the objective quality.

## 1. Introduction

During the past years, along with the rapid development of economy and the improvement of people’s living standard, large quantities of hazardous wastes belonging to different categories are produced in the processes of industrial production and manufacturing, medical services and social life. For example, according to the China Statistical Yearbook on Environment 2018 [1], the number of hazardous wastes produced in China increases from 13.57 million tons in 2008 to 69.36 million tons in 2017 with a compound annual growth rate of 17.7% and the number of hazardous waste types reaches 50. In 2017, 58.3% of hazardous wastes is recycled and reused, 36.8% is treated and disposed and 12.6% is stored. The asbestos wastes, acid wastes and caustic wastes are the top three hazardous waste types in terms of quantity, accounting for more than 20% of hazardous wastes produced in this year. Unlike ordinary wastes, hazardous wastes pose threats to the ecological environment and human health which need to be treated and disposed of in a timely and effective manner (Erkut et al. [2]).

Managing hazardous wastes in a region involves a multi-echelon network with multiple waste facilities including recycling, treatment and disposal centers and multiple procedures with respect to the transportation, recycling, treatment and disposal of hazardous wastes and waste residues. In the regional hazardous waste management network, different facilities and procedures which are interrelated with each other not only generate much logistics cost but also pose a risk to the public and the environment (Erkut et al. [2]). We should design an economically effective and environmentally friendly integrated regional hazardous waste management system by incorporating the location of waste facilities and the transportation/allocation of hazardous wastes/residues simultaneously. Meanwhile, planning a regional hazardous waste management network is a long-term multi-period decision-making problem with a planing horizon lasting 20 to 30 years. During the planning horizon, waste facilities are usually built and put into service sequentially, while the economy, population, production and composition of hazardous wastes in the region are also changing. More importantly, disposal centers are special capacity exhaustible waste facilities whose capacity is limited in their life cycle. The multi-period planing horizon property of the regional hazardous waste management system should be considered to ensure the sustainable development of the system.

This paper focuses on the multi-period network design problem in the long-term design of an integrated regional hazardous waste management system with multiple types of hazardous wastes, waste facilities and treatment technologies. The problem lies on determining the location of recycling, treatment and disposal centers, the technology at located treatment centers and the allocation of hazardous wastes and waste residues between waste origins and waste facilities in each period during the planning horizon. To obtain a practically feasible regional hazardous waste management system, many operational and capacity constraints such as the flow conservation of wastes/residues between origins and facilities, consistence in the location of facilities among periods and capacity of waste facilities should be respected. At the same time, two objectives minimizing the total cost and total risk in the location of waste facilities and the transportation of wastes and residues in the planning horizon are optimized to balance the logistics cost and environmental risk in the design of the system.

The hazardous waste network design problem has received great attention in the literature over the past 30 years as a consequence of the increasing production of hazardous wastes. Existing work on the regional hazardous waste management has mainly addressed the single period static network design problem with either single type of hazardous waste or multiple types of hazardous wastes. Many initial studies focused on the single period hazardous waste network design with single type of hazardous waste due to the seminal work of Zografros and Samara [3]. ReVelle et al. [4], Stowers and Palekar [5], Wyman and Kuby [6] and Boyer et al. [7] developed multi-obejctive location-allocation models of the single period hazardous waste network design with one type of hazardous waste under different structures of networks. Later, Jacobs and Warmerdam [8], Current and Ratick [9], Giannikos [10] and Cappanera et al. [11] solved similar problems but further considered the transportation routes of hazardous wastes and waste residues from waste origins to waste facilities. They formulated multi-objective continuous network flow models to determine the location of waste facilities and the transportation routes of hazardous wastes/waste residues simultaneously.

List and Mirchandani [12] firstly introduced the single period regional hazardous waste network design problem with multiple types of hazardous wastes and treatment technologies. They proposed a multi-objective path-based network design model and solved it with a weighted-sum algorithm. Based on the work of List and Mirchandani [12], much previous research contributed to the same topic by enlarging the considered management system frameworks and respecting more realistic constraints. Alidi [13] provided a goal programming network design model to the regional hazardous waste management system with recycling, treatment and disposal facilities. Emek and Kara [14] treated the location of incinerators in a regional hazardous waste management system with recycling and treatment facilities. Nema and Gupta [15] extended the problem in List and Mirchandani [12] by considering multiple types of hazardous wastes and waste facilities and described the comparability requirements between hazardous wastes and treatment technologies. Alumur and Kara [16] considered the location of treatment and disposal centers and provided a linear constraints for the comparability between wastes and technologies defined in Nema and Gupta [15].

Samanlioglu [17] defined a hazardous waste network design problem incorporating the location of recycling, treatment and disposal centers simultaneously. Ghezavati and Morakabatchian [18] extended the model in Samanlioglu [17] by further optimizing the vehicle routes from waste origins to collection centers. Yu and Solvang [19] developed a network design model including a policy instrument for promoting waste recycling into the regional hazardous waste management system. Zhao et al. [20] formulated a general-purpose multi-objective single period hazardous waste location-transportation model which can take many previous models as special cases. Yilmaz et al. [21] built a more realistic regional hazardous waste management system in which 7 wastes/residues classifications with different treatment procedures were considered. Rabbani et al. [22] further determined the location of separation centers in the design of regional hazardous waste management systems. Aydemir-Karadag [23] considered the profit derived from the polluter pays and electricity sales and formulated a profit-oriented network design model to the hazardous waste management.

Recently, several studies attempted to extend existing hazardous waste network design models into location-routing models in which the vehicle routes from waste origins to certain waste facilities are explicitly optimized. Zhao and Verter [24] formulated a bi-objective location-routing model for a used-oil management system with storage and integrated facilities. The vehicle routes from waste origins to storage facilities were jointly determined with the location of storage and integrated facilities. Zhao and Zhu [25] discussed a location-routing problem in a explosive waste recycling system with collection and recycling centers. A multi-depot vehicle routing model was established to determine the vehicle routes from waste origins to collection centers and those between collection and recycling centers. Zhao and Ke [26] extended the model in Zhao and Zhu [25] by further incorporating the inventory decision and associated inventory risk at collection centers for explosive waste recycling. Asgari et al. [27] presented a multi-objective location-routing model in a hazardous waste management system with treatment and disposal centers to optimize the location of waste facilities and vehicle routes from waste origins to treatment centers. Rabbani et al. [28] improved the formulation proposed in Samanlioglu [17] by routing vehicles to collect hazardous wastes at generation nodes and waste residues generated at different waste facilities.

Multi-period facility location is an active topic in the literature and has a wide range of applications such as in the areas of logistics and supply chain. Interested readers could refer to Nickel and da Gama [29] for a relatively comprehensive review and modeling discussion. However, few previous studies investigated the multi-period network design in regional hazardous waste management systems. Melachrinoudis et al. [30] considered the multi-period location of landfills in a hazardous waste management system with only landfill facilities. A multi-objective model was formulated and solved by the weighted-sum approach. Srivastava and Nema [31] presented a new multi-objective model for the multi-period location of solid waste disposal facilities. A new disposal risk estimation method was proposed and a realistic case study was conducted. Nema and Modak [32] analyzed the importance and necessity of designing the regional hazardous waste management system from a multi-period perspective. A conceptual multi-period network design model was formulated for a system with treatment and disposal centers. Najm et al. [33] established a single objective multi-period network design model for a regional solid waste management system with treatment and disposal centers. The feasibility and efficiency of the model and the impacts of important parameters were illustrated by a case study in Najm et al. [34]. Mitropoulos et al. [35] treated the multi-period network design problem in an integrated solid waste management system with transfer stations, treatment facilities and landfills. A single objective multi-period location-allocation model was established and strengthened by introducing valid inequalities.

Thus, the multi-period network design incorporating the location of waste facilities and the allocation of wastes/residues in each period of the planning horizon has not been completely investigated. Meanwhile, there are several inapplicable issues in previous work on the single period network design for hazardous wastes and multi-period network design for ordinary freights. Firstly, a majority of existing work focuses on the single period network design in regional hazardous waste management systems with different system frameworks and waste facilities. There is no literature considering the location of recycling, treatment and disposal centers and the allocation of wastes/residues between waste origins and these waste facilities from a long-term perspective. Then, in most related work on the multi-period facility location for ordinary freights, the capacity of facilities in the planning horizon is unlimited. However, the capacity of hazardous waste landfills is an exhaustible resource. Each landfill has an upper capacity in its life cycle limited by the available landfill volume. The landfills should be closed in the planning horizon once their life cycle capacity has been reached. Besides, existing studies on the multi-period location of hazardous waste facilities mainly concern how to open new facilities. As mentioned, the regional hazardous waste management is a dynamic decision-making process. In the planning horizon, following the variation in the production of wastes, we should not only decide whether and how to open new waste facilities but also consider whether and how to close existing waste facilities without available capacity or cost/risk attractiveness.

In light of the shortages of related works, in this paper we aim at presenting a theoretically rigorous multi-objective model and a practically feasible multi-objective algorithm for the multi-period network design problem in regional hazardous waste management systems with multiple types of hazardous wastes, waste facilities and treatment technologies. Compared with exiting studies, the contributions of our paper are mainly twofold. Firstly, we formulate the multi-period hazardous waste network design problem as a bi-objective mixed integer linear programming model to minimize the total cost and total risk in the location and transportation procedures simultaneously. A unified modeling strategy is adopted to describe the closing of existing waste facilities and the opening of new waste facilities. Secondly, by exploiting the characteristics of the problem, an augmented ε-constraint algorithm is customized to solve the proposed bi-objective network design model and to find highly qualified representative non-dominated solutions for decision-makers.

The rest of the paper is structured as follows. Section 2 provides a formal description and assumption of the problem investigated in the paper. In Section 3, we formulate the problem as a bi-objective location-allocation model incorporating all decisions, objectives and constraints. An augmented ε-constraint algorithm is developed to solve the proposed bi-objective model in Section 4. In Section 5, a realistic case is used to test the proposed multi-objective model and algorithm, and compare the multi-period network design decision with the single-period decision. Finally, Section 6 summarizes the main work in the paper and provides directions for future research.

## 2. Problem Description

**Figure 1:**
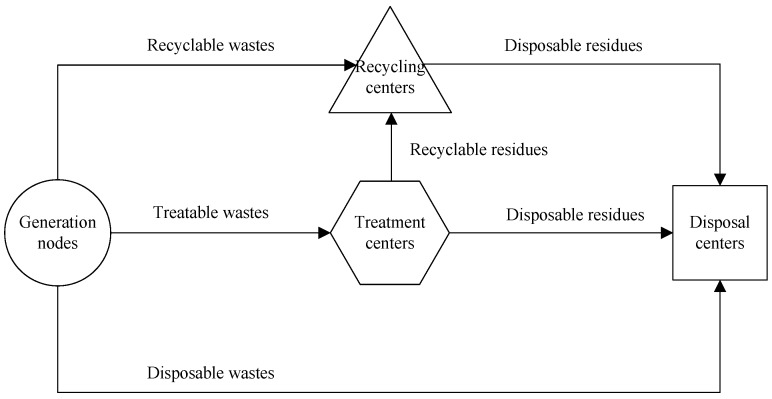
Regional hazardous waste management systems.

We consider a regional hazardous waste management system with multiple types of hazardous wastes, waste facilities and treatment technologies as shown in Figure 1. The system operates three types of waste facilities including recycling centers, treatment centers and disposal centers and routes six bunches of waste/residue flows from generation nodes to waste facilities and between these waste facilities. Generation nodes produce different types of hazardous wastes per each planning period which are assumed to be classified into recyclable, treatable and disposal wastes. The recyclable wastes are directly transported to recycling centers for the reusing and recycling of resources. It is required that the treatable wastes should be firstly transported to and treated at treatment centers by comparable technologies such that the hazardous characteristics of these wastes can be reduced. The disposal wastes are transported to and disposed at disposal centers always using security landfills.

There are many treatment technologies such as the chemical, solidification and incineration technology suitable for different types of hazardous wastes at treatment centers, see LaGrega et al. [36]. Treatment centers use comparable technologies to transform hazardous wastes into waste residues which are supposed to be divided into recyclable and disposable residues. The recyclable residues are transported to and reused at recycling centers, while the disposal residues are transported to and disposed at disposal centers. Finally, the remaining hazardous wastes and waste residues after the reusing at recycling centers are transported to and disposed at disposal centers. Then, the management of hazardous wastes and waste residues in a region is completed.

Given the production of hazardous wastes, candidate waste facilities and related cost, risk and capacity parameters in each period of the planning horizon, the multi-period network design problem in a regional hazardous waste management system is to determine the location of recycling centers, treatment centers (including the treatment technologies) and disposal centers and the allocation/transportation of six hazardous wastes/waste residues from generation nodes to waste facilities and between these waste facilities, such that the wastes and residues produced in each period are treated properly and the opening and closing of each waste facility is consistent among periods, while the total cost and total risk incurred in the location and transportation procedures of the whole planning horizon is minimized. More specifically, the multi-period network design problem should answer the following questions in each period of the planning horizon:

- Which existing recycling centers, treatment centers and disposal centers are closed.
- Which new recycling centers, treatment centers with which types of treatment technologies and disposal centers are opened.
- How to transport or allocate the recyclable, treatable and disposable hazardous wastes at generation nodes among recycling centers, treatment centers and disposal centers, respectively.
- How to transport or allocate the recyclable and disposable waste residues at treatment centers among recycling centers and disposal centers, respectively.
- How to transport or allocate the disposable waste residues at recycling centers among disposal centers.

We design a regional hazardous waste management system from a strategic long-term decision-making perspective and focus on the location of different types of waste facilities and the allocation of different types of wastes/residues among waste facilities. To this end, the detailed treatment and disposal processes of wastes and residues at waste facilities and the transportation procedures of wastes and residues between generation nodes and waste facilities are not considered explicitly. To facilitate the next approach description, the following assumptions are introduced.

- The planning horizon is discretized into a limited number of periods. The related cost, risk and capacity parameters in each period is deterministic. The cost in each period is uniformly discounted at the beginning of the planning horizon using a discount rate determined by the inflation rate divided by the nominal interest rate.
- The existing waste facilities remained from the last planning horizon are not included into the current planning horizon if they have no attractiveness in the aspects of capacity, cost and risk. Due to this selection, any existing waste facilities chosen into the current planning horizon must be operated by at least one period.
- Each existing waste facility can be closed at the end of a period. It cannot be reopened during the remaining of the planing horizon once it is closed at a period. If an existing waste facility is closed at the last period of the planning horizon, it means that this waste facility is operated in the whole planning horizon.
- Each new waste facility is opened at the beginning of a period. It cannot be closed during the remaining of the planing horizon once it is opened at a period.

## 3. Multi-Objective Model

### 3.1. Notations

In this section, we formulate the multi-period network design in regional hazardous waste management systems as a bi-objective optimization model. The notations for the sets, parameters and decision variables to be used in the model are defined and explained as follows.

*Sets*:

$R^{e}\left(T^{e},D^{e}\right)$: set of existing recycling (treatment, disposal) centers.

$R^{n}\left(T^{n},D^{n}\right)$: set of candidate new recycling (treatment, disposal) centers.

$R\left(T,D\right)$: set of existing and new recycling (treatment, disposal) centers indexed by *r* (*t* or *d* ).

*L*: set of treatment technologies indexed by *l*.

*H*: set of hazardous wastes indexed by *h*.

*W*: set of types of hazardous wastes indexed by *w*.

*P*: set of discretized periods indexed by *p* in the planning horizon.

$P_{p}^{1}$: set of effective operation periods for waste facilities which are closed at the end of period p∈P or opened at the beginning of period p∈P. According to the problem assumptions, for an existing waste facility, if it is closed at the end of period *p*, it has been operating in and before period *p*, that is $P_{p}^{1}=\left\{p^{\prime }\in P,\;p^{\prime }\leq p\right\}$. For a new waste facility, if it is opened at the beginning of period *p*, it should keep in operation in and after period *p*, that is, $P_{p}^{1}=\left\{p^{\prime }\in P,\;p^{\prime }\geq p\right\}$.

$P_{p}^{2}$: set of relevant periods for waste facilities indicating that the facilities are operated in period p∈P. If an existing waste facility is closed at the end of period *p* or another period after *p*, it is operated in period *p*. Thus, we have $P_{p}^{2}=\left\{p^{\prime }\in P,\;p^{\prime }\geq p\right\}$. If a new waste facility is opened at the beginning of period *p* or another period before *p*, it is operated in period *p*. It holds $P_{p}^{2}=\left\{p^{\prime }\in P,\;p^{\prime }\leq p\right\}$.

*Parameters*:

$c_{hrp}^{1}$, $c_{htp}^{2}$ and $c_{hdp}^{3}$: cost of transporting one unit of hazardous waste h∈H from the generation node to recycling center r∈R, treatment center t∈T and disposal center d∈D in period p∈P, respectively.

$c_{trp}^{4}$ and $c_{tdp}^{5}$: cost of transporting one unit of waste residues from treatment center t∈T to recycling center r∈R and disposal center d∈D in period p∈P, respectively.

$c_{rdp}^{6}$: cost of transporting one unit of waste residues from recycling center r∈R to disposal center d∈D in period p∈P.

$g_{hrp}^{1}$, $g_{htp}^{2}$ and $g_{hdp}^{3}$: risk of transporting one unit of hazardous waste h∈H from the generation node to recycling center r∈R, treatment center t∈T and disposal center d∈D in period p∈P, respectively.

$g_{trp}^{4}$ and $g_{tdp}^{5}$: risk of transporting one unit of waste residues from treatment center t∈T to recycling center r∈R and disposal center d∈D in period p∈P, respectively.

$g_{rdp}^{6}$: risk of transporting one unit of waste residues from recycling center r∈R to disposal center d∈D in period p∈P.

$f_{rp}^{1}$, $o_{rp}^{1}$, $v_{rp}^{1}$ and $s_{rp}^{1}$: fixed opening/closing cost, fixed operating cost, unit recycling cost and unit recycling risk at recycling center r∈R in period p∈P, respectively.

$f_{tlp}^{2}$, $o_{tlp}^{2}$, $v_{tlp}^{2}$ and $s_{tlp}^{2}$: fixed opening/closing cost, fixed operating cost, unit treatment cost and unit treatment risk at treatment center t∈T with technology l∈L in period p∈P, respectively.

$f_{dp}^{3}$, $o_{dp}^{3}$, $v_{dp}^{3}$ and $s_{dp}^{3}$: fixed opening/closing cost, fixed operating cost, unit disposal cost and unit disposal risk at disposal center d∈D in period p∈P, respectively. The fixed closing cost of existing waste facilities in the last period of the planning horizon is set to 0 for the modeling purpose.

Note that there are many methods in the literature to measure the transportation risk of hazardous wastes and the location risk of waste facilities, please refer to Erkut et al. [2] for discussion. Here, the transportation and location risk are both measured by the traditional risk method, which has been widely used in existing studies, such as Nema and Gupta [15], Alumur and Kara [16], Samanlioglu [17] and Zhao et al. [20]. The unit of the risk is people · ton.

$\lambda_{rp}$: average recycling rate of hazardous wastes and waste residues at recycling center r∈R in period p∈P.

$\eta_{lwp}$: percentage of residues in waste type w∈W after being treated by technology l∈L in period p∈P.

$\kappa_{lwp}$ and $\sigma_{lwp}$: recyclable rate and disposable rate of residues in waste type w∈W after being treated by technology l∈L in period p∈P, respectively.

$\delta_{lw}$: 1 if waste type w∈W is comparable with technology l∈L, 0 otherwise.

$\tau_{h}$: type of hazardous waste h∈H.

$q_{hp}$, $\alpha_{hp}$, $\beta_{hp}$ and $\gamma_{hp}$: amount, recyclable rate, treatable rate and disposable rate of hazardous waste h∈H in period p∈P, respectively.

$a_{rp}^{1}$ and $b_{rp}^{1}$: minimal recycling workload and maximum recycling capacity of recycling center r∈R in period p∈P, respectively.

$a_{tlp}^{2}$ and $b_{tlp}^{2}$: minimal treatment workload and maximum treatment capacity of treatment center t∈T with technology l∈L in period p∈P, respectively.

$a_{dp}^{3}$ and $b_{dp}^{3}$: minimal disposal workload and maximum disposal capacity of disposal center d∈D in period p∈P, respectively.

$b_{d}^{\max}$: maximum capacity of disposal center d∈D in its life cycle. The life cycle capacity of existing waste facilities should subtract its used capacity in the last planning horizon.

*Decision variables*:

$x_{hrp}^{1}$, $x_{htlp}^{2}$ and $x_{hdp}^{3}$: amount of recyclable, treatable and disposable portions in hazardous waste h∈H recycled at recycling center r∈R, treated at treatment center t∈T with technology l∈L, and disposed at disposal center d∈D in period p∈P, respectively.

$x_{trp}^{4}$ and $x_{tdp}^{5}$: amount of recyclable and disposable waste residues in treatment center t∈T recycled at recycling center r∈R and disposed at disposal center d∈D in period p∈P, respectively.

$x_{rdp}^{6}$: amount of disposable waste residues in recycling center r∈R disposed at disposal center d∈D in period p∈P.

$y_{rp}^{1}$: amount of hazardous wastes and waste residues recycled at recycling center r∈R in period p∈P.

$y_{tlwp}^{4}$: amount of hazardous wastes in type w∈W treated at treatment center t∈T with technology l∈L in period p∈P.

$y_{tlp}^{2}$: amount of all types of hazardous wastes treated at treatment center t∈T with technology l∈L in period p∈P.

$y_{dp}^{3}$: amount of waste residues disposed at disposal center d∈D in period p∈P.

$z_{rp}^{1}$: 0–1 variables, 1 if existing recycling center $r\in R^{e}$ is closed at the end of period p∈P or new recycling center $r\in R^{n}$ is opened at the beginning of period p∈P, 0 otherwise.

$z_{tlp}^{2}$: 0–1 variables, 1 if existing treatment center $t\in T^{e}$ using technology l∈L is closed at the end of period p∈P or new treatment center $t\in T^{n}$ using technology l∈L is opened at the beginning of period p∈P, 0 otherwise.

$z_{dp}^{3}$: 0–1 variables, 1 if existing disposal center $d\in D^{e}$ is closed at the end of period p∈P or new disposal center $d\in D^{n}$ is opened at the beginning of period p∈P, 0 otherwise.

### 3.2. Model Formulation

Using the above notations, the multi-period network design problem in regional hazardous waste management systems (MPND-RHWMS) can be formulated as a bi-objective optimization model as follows:

(MPND-RHWMS)

(1) $$\begin{array}{cc} \min\;z_{1}= & \sum_{h\in H}\sum_{r\in R}\sum_{p\in P}c_{hrp}^{1}x_{hrp}^{1}+\sum_{h\in H}\sum_{t\in T}\sum_{l\in L}\sum_{p\in P}c_{htp}^{2}x_{htlp}^{2}+\sum_{h\in H}\sum_{d\in D}\sum_{p\in P}c_{hdp}^{3}x_{hdp}^{3}\\ & +\sum_{t\in T}\sum_{r\in R}\sum_{p\in P}c_{trp}^{4}x_{trp}^{4}+\sum_{t\in T}\sum_{d\in D}\sum_{p\in P}c_{tdp}^{5}x_{tdp}^{5}+\sum_{r\in R}\sum_{d\in D}\sum_{p\in P}c_{rdp}^{6}x_{rdp}^{6}\\ & +\sum_{r\in R}\sum_{p\in P}\sum_{p^{\prime }\in P_{p}^{1}}\left(f_{rp}^{1}+o_{rp^{\prime }}^{1}\right)z_{rp}^{1}+\sum_{t\in T}\sum_{l\in L}\sum_{p\in P}\sum_{p^{\prime }\in P_{p}^{1}}\left(f_{tlp}^{2}+o_{tlp^{\prime }}^{2}\right)z_{tlp}^{2}\\ & +\sum_{d\in D}\sum_{p\in P}\sum_{p^{\prime }\in P_{p}^{1}}\left(f_{dp}^{3}+o_{dp^{\prime }}^{3}\right)z_{dp}^{3}\\ & +\sum_{r\in R}\sum_{p\in P}v_{rp}^{1}y_{rp}^{1}+\sum_{t\in T}\sum_{l\in L}\sum_{p\in P}v_{tlp}^{2}y_{tlp}^{2}+\sum_{d\in D}\sum_{p\in P}v_{dp}^{3}y_{dp}^{3} \end{array}$$

(2) $$\begin{array}{cc} \min\;z_{2}= & \sum_{h\in H}\sum_{r\in R}\sum_{p\in P}g_{hrp}^{1}x_{hrp}^{1}+\sum_{h\in H}\sum_{t\in T}\sum_{l\in L}\sum_{p\in P}g_{htp}^{2}x_{htlp}^{2}+\sum_{h\in H}\sum_{d\in D}\sum_{p\in P}g_{hdp}^{3}x_{hdp}^{3}\\ & +\sum_{t\in T}\sum_{r\in R}\sum_{p\in P}g_{trp}^{4}x_{trp}^{4}+\sum_{t\in T}\sum_{d\in D}\sum_{p\in P}g_{tdp}^{5}x_{tdp}^{5}+\sum_{r\in R}\sum_{d\in D}\sum_{p\in P}g_{rdp}^{6}x_{rdp}^{6}\\ & +\sum_{r\in R}\sum_{p\in P}s_{rp}^{1}y_{rp}^{1}+\sum_{t\in T}\sum_{l\in L}\sum_{p\in P}s_{tlp}^{2}y_{tlp}^{2}+\sum_{d\in D}\sum_{p\in P}\sum_{p^{\prime }\geq p}s_{dp^{\prime }}^{3}y_{dp}^{3} \end{array}$$

(3) $$s.t.\;\sum_{r\in R}x_{hrp}^{1}=\alpha_{hp}q_{hp}\;\forall h\in H,p\in P$$

(4) $$\sum_{t\in T}\sum_{l\in L}x_{htlp}^{2}=\beta_{hp}q_{hp}\;\forall h\in H,p\in P$$

(5) $$\sum_{d\in D}x_{hdp}^{3}=\gamma_{hp}q_{hp}\;\forall h\in H,p\in P$$

(6) $$\sum_{r\in R}x_{trp}^{4}=\sum_{l\in L}\sum_{w\in W}\eta_{lwp}\kappa_{lwp}y_{tlwp}^{4}\;\forall t\in T,p\in P$$

(7) $$\sum_{d\in D}x_{tdp}^{5}=\sum_{l\in L}\sum_{w\in W}\eta_{lwp}\sigma_{lwp}y_{tlwp}^{4}\;\forall t\in T,p\in P$$

(8) $$\sum_{d\in D}x_{rdp}^{6}=\left(1-\lambda_{rp}\right)y_{rp}^{1}\;\forall r\in R,p\in P$$

(9) $$\sum_{h\in H}x_{hrp}^{1}+\sum_{t\in T}x_{trp}^{4}=y_{rp}^{1}\;\forall r\in R,p\in P$$

(10) $$\sum_{\left.h\in H\;\right|\;\tau_{h}=w}x_{htlp}^{2}=y_{tlwp}^{4}\;\forall t\in T,l\in L,w\in W,p\in P$$

(11) $$\sum_{w\in W}y_{tlwp}^{4}=y_{tlp}^{2}\;\forall t\in T,l\in L,p\in P$$

(12) $$\sum_{h\in H}x_{hdp}^{3}+\sum_{t\in T}x_{tdp}^{5}+\sum_{r\in R}x_{rdp}^{6}=y_{dp}^{3}\;\forall d\in D,p\in P$$

(13) $$a_{rp}^{1}\sum_{p^{\prime }\in P_{p}^{2}}z_{rp^{\prime }}^{1}\leq y_{rp}^{1}\leq b_{rp}^{1}\sum_{p^{\prime }\in P_{p}^{2}}z_{rp^{\prime }}^{1}\;\forall r\in R,p\in P$$

(14) $$a_{tlp}^{2}\sum_{p^{\prime }\in P_{p}^{2}}z_{tlp^{\prime }}^{2}\leq y_{tlp}^{2}\leq b_{tlp}^{2}\sum_{p^{\prime }\in P_{p}^{2}}z_{tlp^{\prime }}^{2}\;\forall t\in T,p\in P$$

(15) $$a_{dp}^{3}\sum_{p^{\prime }\in P_{p}^{2}}z_{dp^{\prime }}^{3}\leq y_{dp}^{3}\leq b_{dp}^{3}\sum_{p^{\prime }\in P_{p}^{2}}z_{dp^{\prime }}^{3}\;\forall d\in D,p\in P$$

(16) $$\sum_{p\in P}y_{dp}^{3}\leq b_{d}^{\max}\;\forall d\in D$$

(17) $$\sum_{p\in P}z_{rp}^{1}=1\;\forall r\in R^{e}$$

(18) $$\sum_{p\in P}z_{rp}^{1}\leq 1\;\forall r\in R^{n}$$

(19) $$\sum_{p\in P}z_{tlp}^{2}=1\;\forall t\in T^{e},l\in L$$

(20) $$\sum_{p\in P}z_{tlp}^{2}\leq 1\;\forall t\in T^{n},l\in L$$

(21) $$\sum_{p\in P}z_{dp}^{3}=1\;\forall d\in D^{e}$$

(22) $$\sum_{p\in P}z_{dp}^{3}\leq 1\;\forall d\in D^{n}$$

(23) $$x_{htlp}^{2}\leq b_{tlp}^{2}\delta_{l\tau_{h}}\;\forall h\in H,t\in T,l\in L,p\in P$$

(24) $$y_{tlwp}^{4}\leq b_{tlp}^{2}\delta_{lw}\;\forall t\in T,l\in L,w\in W,p\in P$$

(25) $$x_{hrp}^{1},x_{htlp}^{2},x_{hdp}^{3},x_{trp}^{4},x_{tdp}^{5},x_{rdp}^{6}\geq 0\;\forall h,r,t,l,d,p$$

(26) $$y_{rp}^{1},y_{tlwp}^{4},y_{tlp}^{2},y_{dp}^{3}\geq 0\;\forall r,t,l,w,d,p$$

(27) $$z_{rp}^{1},z_{tlp}^{2},z_{dp}^{3}\in \left\{0,1\right\}\;\forall r,t,l,d,p$$

Objective (1) including twelve parts minimizes the total cost in the transportation and location procedures of the whole planning horizon. The first three parts are the transportation cost of hazardous wastes from generation nodes to recycling centers, treatment centers and disposal centers, respectively. The transportation cost of waste residues from treatment centers to recycling centers and disposal centers are given by the fourth and fifth parts, respectively. The sixth part provides the transportation cost of waste residues from recycling centers to disposal centers. The seventh to ninth parts calculate the fixed cost of locating and operating recycling centers, treatment centers and disposal centers, respectively. Based on our modeling strategy, the fixed cost of both existing and new waste facilities can be correctly counted using the same expression with set $P_{p}^{1}$. The last three parts determine the variable process cost at recycling centers, treatment centers and disposal centers, respectively.

Objective (2) is to minimize the total risk in the regional hazardous waste management system. This objective can be understood analogously following Objective (1). There are two notices. The one is that there is only variable process risk at recycling centers, treatment centers and disposal centers depending on the workload at these waste facilities. The another is that the waste residues disposed at a disposal center in period *p* will be remained at the center, incurring risk at the center in the following periods of the planning horizon, see the last part of Objective (2).

Constraints (3) to (5) are the flow conservation for the recyclable portion, treatable portion and disposable portion of hazardous wastes among recycling centers, treatment centers and disposal centers in each period, respectively. Constraints (6) and (7) specify the flow conservation for the recyclable portion and disposable portion of waste residues at treatment centers among recycling centers and disposal centers in each period, respectively. Constraints (8) give the flow conservation for the disposable waste residues at recycling centers among disposal centers in each period. Constraints (9) determine the workload in each period at recycling centers where the wastes are from both generation nodes and treatment centers. Constraints (10) calculate the workload per waste type in each period at treatment centers, while Constraints (11) count the total workload in each period at treatment centers. Constraints (12) compute the workload in each period at disposal centers at which the wastes from generation nodes, treatment centers and recycling centers are all disposed.

Constraints (13) guarantee that the workload in each period at recycling centers is not less than the minimal recycling workload and is not greater than the maximum recycling capacity. Constraints (14) and (15) are similar constraints for treatment centers and disposal centers, respectively. With the definition of set $P_{p}^{2}$, the minimal workload and maximum capacity constraints of both existing and new waste facilities can be modeled uniformly. Constraints (16) ensure that the total workload in the planning horizon at disposal centers should be less than the life cycle capacity. Constraints (17) and (18) are the location consistence of recycling centers. Constraints (17) assure that existing recycling centers are operated by at least one period and they cannot be reopened once they are closed. Constraints (18) make sure that new recycling centers can be opened by at most once and they should be operated until the end of the planning horizon once they are opened. Constraints (19) and (20) are the location consistence of treatment centers, while Constraints (21) and (22) are those for disposal centers. Constraints (23) and (24) are the waste-technology comparability requirement with which hazardous wastes can only be treated by comparable technologies. Constraints (25) to (27) specify the integer and non-negative requirements of decision variables.

## 4. Augmented ε-Constraint Algorithm

Our problem (MPND-RHWMS) is obviously NP-hard as its single-objective version can be reduced to the capacitated multi-period facility location problem. The latter problem has been proved to be NP-hard in many existing works, see Nickel and da Gama [29]. Thus, our problem is NP-hard. Meanwhile, the problem is inheritably a multi-objective mixed integer programming problem which should be solved to find a set of representative non-dominated solutions based on which decision-makers can choose the attractive solutions according to the conflicts of objectives and personal preferences. The original problem holds solving difficulties due to the problem complexity and multiple objectives. However, our proposed model is a mixed integer linear programming one. Any single objective of the model can be solved to optimality within a reasonable time by commercial solvers for practical-sized problems. Meanwhile, there are several efficient multi-objective optimization algorithms in the literature which can be used to solve multi-objective mixed integer programming problems once a compact multi-objective optimization model is given. Besides, as mentioned, our problem is a strategic long-term decision-making problem in which the computation time is not strict. Thus, according to our observations, we utilize the augmented ε-constraint algorithm which are recognized and widely used in related studies (e.g., Yu and Solvang [19], Zhao et al. [20]) to solve the multi-period network design in regional hazardous waste management systems.

The augmented ε-constraint algorithm initialized in Haimes et al. [37] and improved by Mavrotas [38] and Mavrotas and Florios [39] is one of the theoretically and computationally attractive multi-objective mixed integer linear programming algorithms till now, which has been widely used to solve the multi-objective optimization problems in the areas of production, manufacturing, transportation and logistics. The basic principle of the algorithm is to optimize only one objective of the considered multi-objective problem and add other objectives into constraints in a form of being not inferior to given values, such that the multi-objective problem is transformed into a single objective one. Based on that, representative non-dominated solutions of the multi-objective problem can be identified by dynamically adjusting the right-hand-side value of constrained objectives and solving the underlying single objective problem to optimality. To avoid the algorithm from producing weak non-dominated solutions when the inequality constraints of constrained objectives are not active, in Mavrotas and Florios [39], additional slackness/surplus variables are introduced to transform the inequality constraints of objectives into equality ones and then these additional variables after being normalized and multiplied by a sufficient constant ρ>0 are augmented into the only objective, such that we can be guaranteed that only non-dominated solutions will be found by the augmented single objective problem. Thus, we customize the augmented ε-constraint algorithm proposed by Mavrotas and Florios [39] to solve our multi-period network design problem.

For simplicity, the original problem (MPND-RHWMS) is denoted as $\min\;z\left(x\right)=\left\{z_{1}\left(x\right),z_{2}\left(x\right)\right\}$, s.t. x∈X, where $z_{1}\left(x\right)$ and $z_{2}\left(x\right)$ are the total cost objective (1) and total risk objective (2), respectively, and *X* represents the feasible region of the problem composed by Constraints (3)–(27). Without loss of generality, in the original problem, we only optimize the total cost objective $z_{1}\left(x\right)$ and add the total risk objective $z_{2}\left(x\right)$ being not inferior to given values into constraints. The solution procedures of the augmented ε-constraint algorithm are described as follows.

*Step 1*: Determine the minimal value $z_{2}^{\min}$, maximum value $z_{2}^{\max}$ and value range $\nu_{2}=z_{2}^{\max}-z_{2}^{\min}$ of the total risk objective $z_{2}\left(x\right)$. Obviously, the minimal value $z_{2}^{\min}$ can be obtained by solving the original problem (MPND-RHWMS) under the total risk objective (2). Let $x_{2}$ be the optimal solution to the original problem under the total risk objective, we have $z_{2}^{\min}=z_{2}\left(x_{2}\right)$. The determination of the maximum value $z_{2}^{\max}$ is relatively complicated. We use a lexicographic method introduced by Mavrotas and Florios [39] to determine the maximum value $z_{2}^{\max}$ of the total risk objective as follows:

(28) $$\begin{array}{cc} z_{2}^{\max} & =\min\;z_{2}\left(x\right)\\ s.t. & z_{1}\left(x\right)=z_{1}^{\min}\\ & x\in X \end{array}$$

*Step 2*: Let the required number of non-dominated solutions be $g_{2}+1$. Then, divide the value range $\nu_{2}$ of the total risk objective into $g_{2}$ even segments each of which the length is ν2ν2g2g2 using $g_{2}+1$ evenly distributed grid points including the points with respect to the minimal value and maximum value of the total risk objective. To this end, we search non-dominated solutions at these $g_{2}+1$ grid points. The index of the grid points is denoted as $k_{2}$. Initialize $k_{2}$= 0 and go to the next step.

*Step 3*: If the current grid point $k_{2}\leq g_{2}$, let the corresponding right-hand-side value of the constrained total risk objective Δ2=z2max−k2ν2ν2g2g2 and find the non-dominated solution at grid point $k_{2}$ by solving the following augmented ε-constraint model. Otherwise, the algorithm is terminated.

(29) $$\begin{array}{cc} \min & \bar{z}=z_{1}\left(x\right)-\rho \mu_{2}/\nu_{2}\\ s.t. & z_{2}\left(x\right)+\mu_{2}=\Delta_{2}\\ & \mu_{2}\geq 0\\ & x\in X \end{array}$$

where $\mu_{2}\geq 0$ is the slackness variable in the constraints of the total risk objective.

*Step 4*: If an optimal solution $\left\{x^{*},\mu_{2}^{*}\right\}$ is found in model (29), then a non-dominated solution $\left(z_{1}\left(x^{*}\right),z_{2}\left(x^{*}\right)\right)$ is obtained at grid point $k_{2}$. Go to the next step. Otherwise, go to Step 6.

*Step 5*: If in the optimal solution of model (29) the slackness variable $\mu_{2}$ satisfies μ2*>ν2ν2g2g2, it means that the next one or several grid points will actually produce the same non-dominated solutions in which only the value of the slackness variable $\mu_{2}$ is decreased by a constant of ν2ν2g2g2. Thus, such a solution will make the next one or several grid points redundant. We can bypass these equivalent grid points and hence accelerate the algorithm. To this end, let $\tau_{2}$ be the bypass coefficient which can be valued as τ2=μ2*g2μ2*g2ν2ν2, update the grid point index $k_{2}=k_{2}+\tau_{2}$ and go back to Step 3.

*Step 6*: Our algorithm gradually strengths the right-hand-side value of the constraints for the total risk objective from the first grid point to the last one. Due to that, if at the current grid point $k_{2}$, model (29) is infeasible, in the following grid points the model also cannot find feasible solutions due to the fact that the right-hand-side value of the total risk objective is more strict. It is unnecessary and invaluable to continue the algorithm. Thus, the algorithm is terminated.

Note that presetting $g_{2}+1$ grid points cannot be guaranteed to find the same number of non-dominated solutions. In case of that the number of obtained non-dominated solutions is not enough, we can increase the number of grid points accordingly and restart the algorithm.

## 5. Case Study

In this section, a realistic case study is conducted to test the feasibility and efficiency of the proposed multi-objective approach. The augmented ε-constraint algorithm is coded in MATLAB 9.0 (MathWorks, Natick, Massachusetts, U.S.) and the underlying single objective model (MPND-RHWMS) is solved by invoking CPLEX 12.8 (IBM, Armonk, New York, U.S.) with parameters being set to the default value. The computation is implemented on a personal computer with Inter Core i7-7700 3.60 GHz CPU, 16.00 GB RAM and Windows 10-64 bits operating system (Microsoft, Redmond, Washington, USA). The parameter ρ in the algorithm is set to $10^{-3}$ according to Mavrotas and Florios [39].

### 5.1. Case Description and Parameter Setting

**Figure 2:**
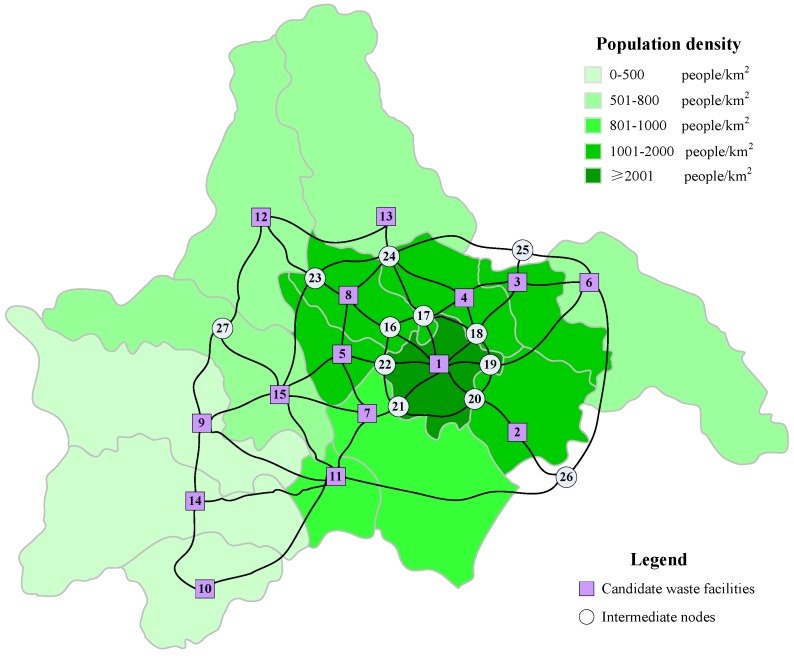
Test hazardous waste management network.

The test case is to design the hazardous waste management system of a big city located in the southwestern of China. The city divided into 15 administrative districts covers an area of 12 thousand square kilometers with a permanent population of 14.4 million. To generate the test case, all administrative districts in the city are selected as the generation nodes of hazardous wastes. Meanwhile, except for the first main urban district, other districts are assumed to be the candidate sites for recycling centers, treatment centers and disposal centers simultaneously. Besides, only the freeways, national and provincial roads between districts can be used to transport hazardous wastes and waste residues. The hazardous waste management network in the test city includes 27 nodes and 54 arcs, as shown in Figure 2. In the Figure, nodes 1–15 are the generation nodes of hazardous wastes and nodes 2–15 are the candidate sites of recycling centers, treatment centers and disposal centers. For any two nodes in the network the shortest path is chosen to transport hazardous wastes and waste residues.

We consider a planning horizon of 25 years to design the test hazardous waste network. The planing horizon is discretized into 5 periods each of which lasts 5 years. To reflect the present value of cash, the cost parameter in each period is discounted at the beginning of the planning horizon using an annual discount rate given by an annual inflation rate of 8% divided by an annual nominal interest rate of 6%. Based on the population and waste prediction, in the planning horizon, the annual population growth rate and annual waste growth rate are both assumed to be 5%. For simplicity, in the following mainly the parameter setting in the first year of the planing horizon is illustrated.

The production of hazardous wastes in the considered city is unknown. To construct the test case, each generation node is assumed to produce three types of hazardous wastes in each year during the planning horizon. Two treatment technologies including the solidification (Tech 1) and incineration (Tech 2) can be used to treat these wastes. The first wastes can only be solidified in which the recyclable, treatable and disposal portions are 0.1, 0.8 and 0.1, respectively. The second wastes for which recycling is forbidden can only be incinerated before the disposal process. The third wastes can either be solidified or incinerated in which the recyclable, treatable and disposal portions are 0.05, 0.9 and 0.05, respectively. The annual amount of each type of hazardous waste at the first year in the city is assumed to be 40 thousand tons. The annual waste amount of each type of hazardous waste at each district is estimated by the ratio of the gross industrial production (GIP) index of the district to that of the city. According to the statistics annual report in the city, the GIP of districts 1–15 is 46.8, 45.7, 19.2, 26.0, 15.2, 5.8, 30.5, 17.8, 4.5, 3.4, 8.9, 4.9, 10.3, 5.7 and 5.7 billion dollars, respectively.

The unit transportation cost of hazardous wastes and waste residues are set as follows. The unit transportation cost of recyclable wastes and residues is 0.5 $/t/km and that of treatable wastes is 1 $/t/km and that of disposable wastes and residues is 0.75 $/t/km. The traditional risk method used in Zhao et al. [20] is adopted to measure the transportation risk. Specifically, the unit transportation risk of wastes/residues on a link = risk potential of wastes/risks × risk probability on the link × risk consequence on the link. The risk potential of recyclable wastes and residues are set to 0.05 and that of treatable wastes is 0.2 and that of disposable wastes and residues are 0.1. The risk probability on a link = 0.36 × length of the link (km) $\times 10^{-6}$. The risk consequence on a link is assessed by the number of people who are exposed within 800 m bandwidth of the link. That is, the risk consequence on a link = 1.6 (km) × length of the link (km) × population density of the link (people/km${}^{2}$).

The cost, capacity and risk parameters of candidate recycling centers, treatment centers and disposal centers are summarized in Table 1, Table 2 and Table 3, respectively. According to Zhao et al. [20], the location risk of a waste facility at a district = location risk probability at the district × location risk consequence at the district. The location risk probability of each type of waste facility at different districts is assumed to the same. The location risk probability of recycling, treatment and disposal centers are provided in Table 1, Table 2 and Table 3, respectively. The location risk consequence at a district is measured by the number of people who are exposed within 2500 m radius of the district. To be specific, the location risk consequence at a district = 6.25 π (km${}^{2}$) × population density of the district (people/km${}^{2}$). The population density of districts is shown in Figure 2.

To make the test case realistic, based on the practical situation, it is given that a recycling center, a treatment center with both solidification and incineration technologies and a disposal center are operated at node 2 at the beginning of the planning horizon and the life cycle capacity of the existing disposal center 2 has be occupied by 30 thousand tons. Besides, the length of the life cycle for disposal centers is set to 20 years and the life cycle capacity of a disposal center is assumed to its maximum disposal capacity per year multiplied by the length of its life cycle.

### 5.2. Computational Results

**Figure 3:**
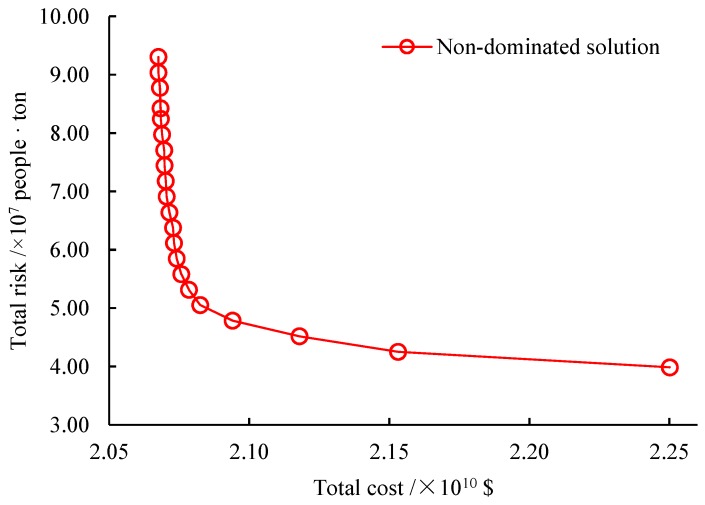
Approximated non-dominated frontier.

We use the augmented ε-constraint algorithm to find 21 representative non-dominated solutions for the test case. The computational results are provided in Table 4. In the Table, the second and third columns are the total cost and total risk for each solution. The fourth column is the computation time of our algorithm to find this solution. The non-dominated frontier of the test case approximated by the 21 solutions is depicted in Figure 3, where the horizontal and vertical axes are the total cost and total risk, respectively.

As seen from Table 4 and Figure 3, in terms of solution quality, the solutions produced by our algorithm are all non-dominated and are distributed uniformly in the objective space. Meanwhile, the non-dominated frontier approximated by these solutions exhibits clearly the trade-off between the total cost and total risk objectives. Specifically, from the first solution with the minimal cost and maximum risk, if the total cost is increased by 0.06%, the total risk will be decreased by 14.3%, meaning that the economic efficiency of risk mitigation (equal to the decrease of total risk divided by the increase of total cost) is 1.06. Note that the bigger the economic efficiency of risk mitigation is, the better risk mitigation is achieved. If the total cost continues to be enlarged by 0.13%, the total risk will be shortened by 16.74%, incurring an economic efficiency of risk mitigation of 0.5. If the total cost is further raised by 0.33%, the total risk will be reduced by 19.91% and the economic efficiency of risk mitigation is 0.19. Until now, the efficiency of increasing the total cost to mitigate the total risk is obvious. However, if the total cost is finally advanced to the maximum value, the economic efficiency of risk mitigation is just 0.01, indicating that increasing the total cost cannot reduce the total risk any more.

Regarding to computation time, due to the complexity of the original problem, the proposed algorithm consumes 3260 s on average to find all non-dominated solutions. The longest computation time to find a solution gets close to 2 h. Even though, as discussed above, there is no strict requirement for the computation time in our problem. Hence, based on the solution quality and computation time, we believe that the augmented ε-constraint algorithm is applicable to solve the strategic multi-period network design in regional hazardous waste management systems.

**Figure 4:**
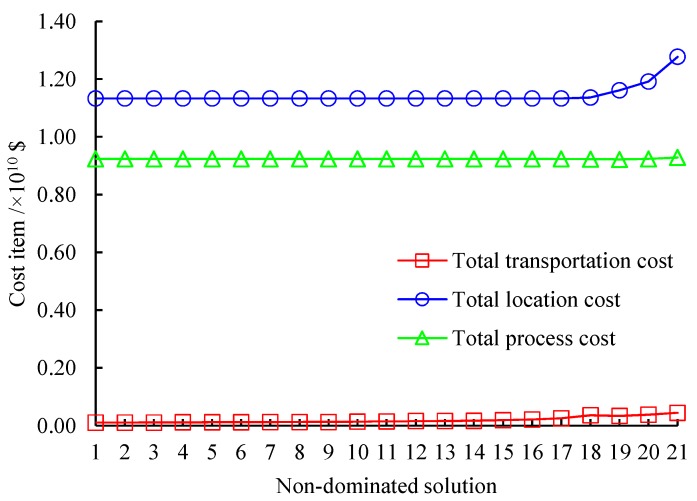
Cost items of non-dominated solutions.

Figure 4 displays the cost items of the 21 non-dominated solutions, where “Total transportation cost” is the sum of the first six parts in Objective (1) and “Total location cost” represents the sum of the seventh to ninth parts in Objective (1) and “Total process cost” expresses the sum of the last three parts in Objective (1). As shown in Figure 4, in all solutions, the proportion of the total location cost is the biggest in the total cost with an average value of 54.81%, followed by that of the total process cost with an average proportion of 44.29%. The total transportation cost is the smallest portion in the total cost and the average proportion is just 0.9%. Meanwhile, following the increase of the total cost, the total transportation cost raises monotonically, while the total location cost and total process cost almost remains stable until the last 4 solutions.

**Figure 5:**
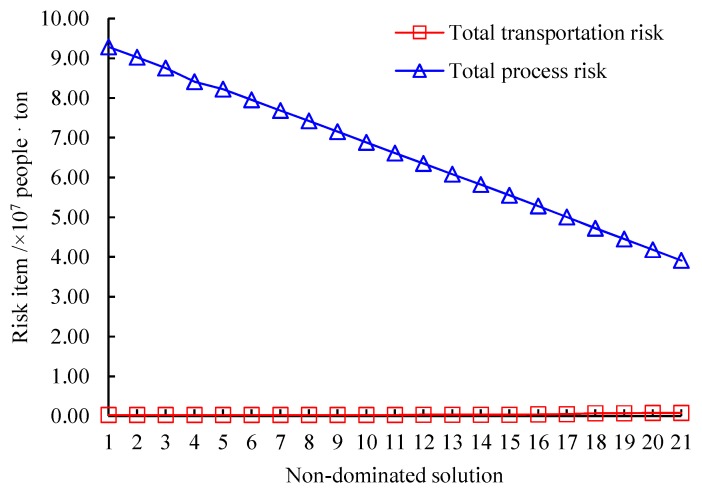
Risk items of non-dominated solutions.

The risk items of the 21 non-dominated solutions is illustrated in Figure 5. There, the sum of the first six parts in Objective (2) is represented by “Total transportation risk” and the sum of the last three parts in Objective (2) is denoted as “Total process risk”. Figure 5 shows that the average proportion of the total process risk in the total risk of all solutions amounts to 99.36%, which is far greater than the average proportion of the total transportation risk which is just 0.64%. Note that with the decrease of the total risk, the total process risk decreases monotonically but the total transportation risk increases monotonically. The reason can be explained by that to mitigate the total risk, large quantities of wastes and residues are transported to and treated at remote waste facilities instead of nearby ones, thus raising the total transportation risk. Based on the observations in Figure 4 and Figure 5 and the fact that the total location cost, total process cost and total process risk all depend on the location of waste facilities, we can see that in the decisions of the design of a regional hazardous waste management system, the location of waste facilities is more important to control the total cost and total risk compared with the transportation/allocation of wastes and residues between generation nodes and waste facilities.

**Figure 6:**
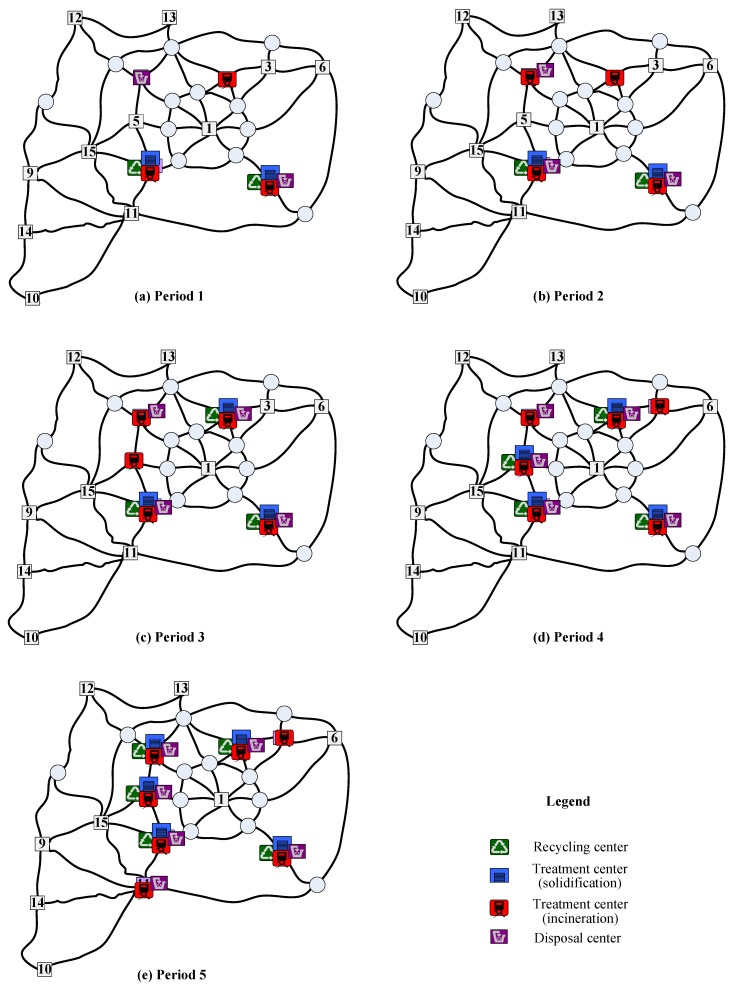
Location in non-dominated solution 1 (minimal total cost).

**Figure 7:**
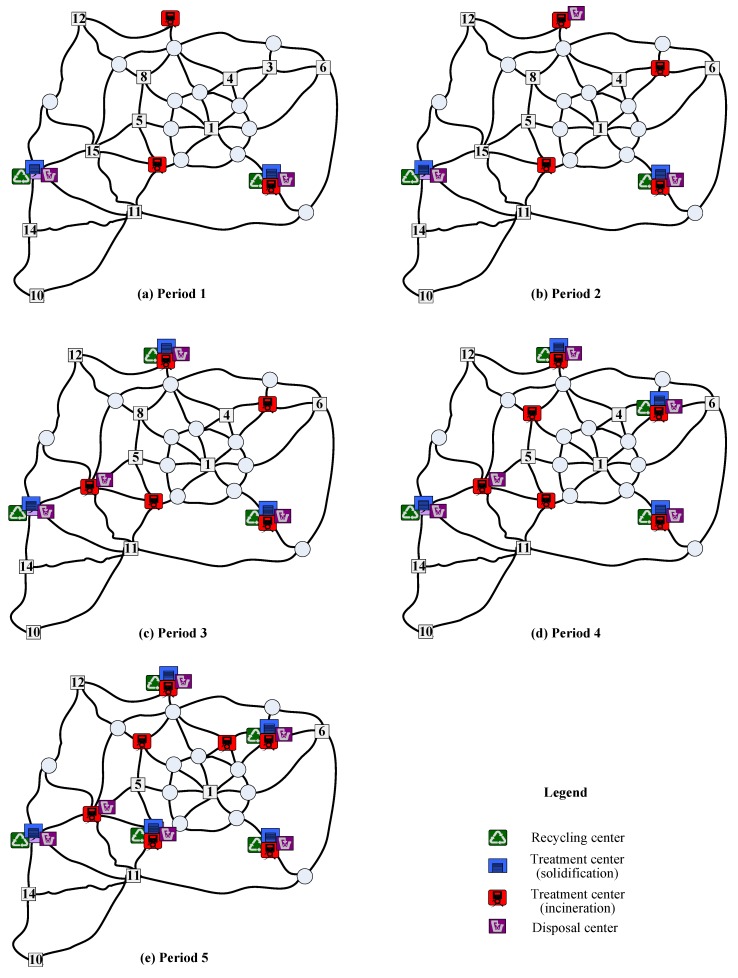
Location in non-dominated solution 2 (balance of two objectives).

**Figure 8:**
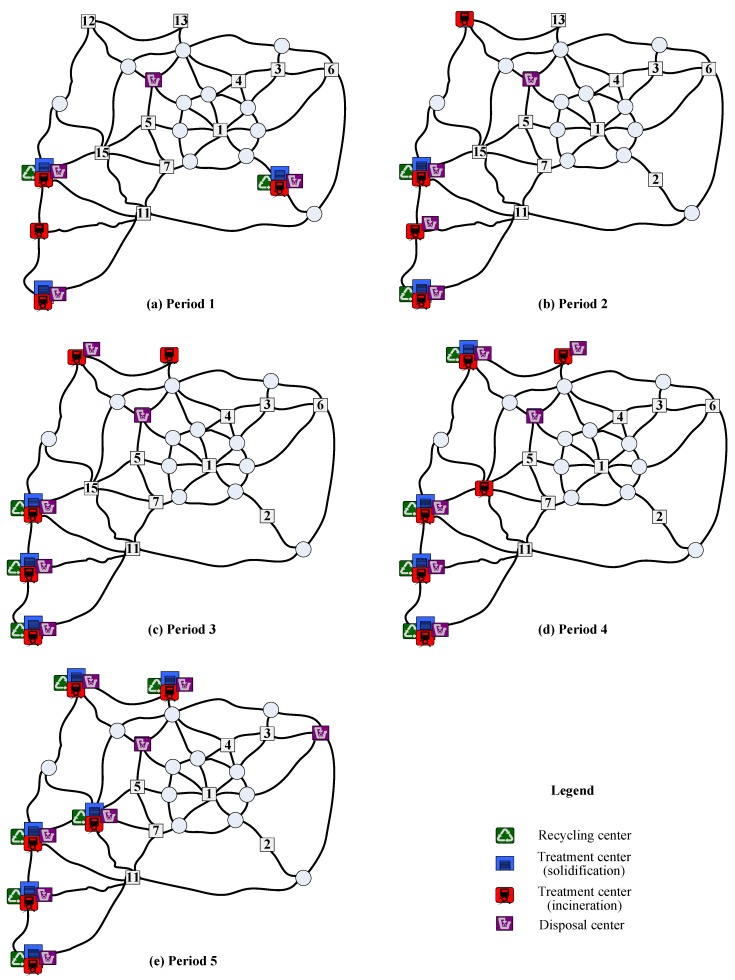
Location in non-dominated solution 3 (minimal total risk).

To reveal the characteristics of non-dominated solutions, the location of waste facilities during the planning horizon in three representative solutions is summarized in Table 5, where solutions 1 and 3 represent the preference for the minimal total cost and minimal total risk respectively, and solution 2 indicates the balance for the two preferences. To ease the understanding, the location of waste facilities in the three non-dominated solutions is also displayed in Figure 6, Figure 7 and Figure 8, respectively. As known from the Table and Figures, different decision preferences will produce different multi-period location of waste facilities. To be specific, if decision-makers want to minimize the total cost, in the whole planning horizon different types of hazardous wastes should be operated at the nodes with a high production of wastes. Especially, integrated waste facilities incorporating recycling, treatment and disposal functions could be located at these nodes (e.g., nodes 2, 4 and 7). If decision-makers like to control the total risk, all types of waste facilities should be intensively set at nodes with a low population in the planning horizon. If conditions feasible, integrated waste facilities could be operated at these nodes such as 9, 10 and 14. If decision-makers prefer to balance the two objectives, they could locate integrated waste facilities at highly accessible nodes in the earlier periods of the planning horizon and then following the increase of waste production and population operate integrated waste facilities at sparsely populated nodes in the later periods of the planning horizon. Observe that node 2 is an existing integrated waste facility. Table 5 reflects that the planners of the test city prefer to locate waste facilities with the aim of minimizing the total cost in the last planning horizon. If they want to mitigate the total risk in the current planning horizon, the effect of closing node 2 is significant but many evaluations should be carefully conducted.

### 5.3. The Value of Multi-Period Optimization

We evaluate the value of multi-period optimization compared to single period optimization on the network design of regional hazardous waste management systems by comparing the representative solutions found by the two approaches. For the comparison, the single period optimization is implemented by using the augmented ε-constraint algorithm to search representative solutions of the original problem over period. In the search of one solution, the waste facilities operated in a period will be taken as existing waste facilities in the next period if they still satisfy the minimal workload and maximum capacity requirements. The solutions found in all periods are combined to be one representative solution of the original problem. It should be noted that the solutions produced by the defined single period optimization could be not the non-dominated solutions of the original problem.

The proposed single period optimization approach can find 21 representative solutions for the original problem very quickly. The average computation time is less than 5 s. The location of waste facilities in the three solutions with respect to minimal total cost, minimal total risk and balance between cost and risk are provided in Table 6. From the Table we can see that the three representative solutions obtained by the single period optimization are all dominated ones to the original problem. Actually, it is theoretically difficult to guarantee that the solutions under single period optimization are still non-dominated. Compared with the single period optimization, multi-period optimization can reduce the minimal total cost by 4.62%. Even the improvement rate is not attractive, the saved money amounts to 1 billion. Meanwhile, the minimal total risk could be mitigated by 9.82%. Thus, conducting the multi-period network design is important and beneficial to obtain economically effective, environmentally friendly and sustainable regional hazardous waste management systems.

### 5.4. Sensitivity Analysis

**Figure 9:**
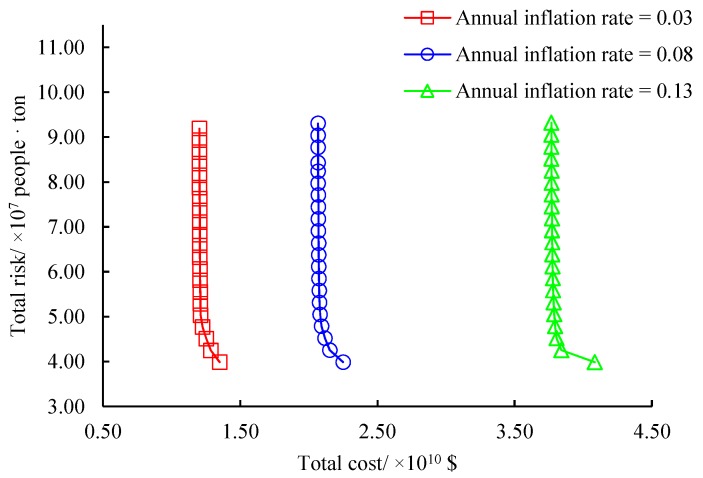
Effects of annual inflation rate.

We first analyze the effects of annual inflation rate on the original problem. In our problem, a discount rate is introduced to discount the cost parameters in all periods at the beginning of the planning horizon. It is defined that annual discount rate = annual inflation rate ÷ annual nominal interest rate. According to the economy development in the test city, three values of annual inflation rates are designed which are 0.03, 0.08 and 0.13, respectively. For each value, maintain other parameters unchanged in the original problem and use our algorithm to construct the non-dominated frontier approximated by 21 solutions. Sensitivity analysis results are shown in Figure 9. As observed, the annual inflation rate does not impact the total risk since the adopted risk measurement is independent of this parameter. Meanwhile, the structure of the three approximated non-dominated frontiers is similar under different annual inflation rates. However, the variation trend of the total cost is different. The average increase of the total cost when the annual inflation rate is from 0.08 to 0.13 is nearly twice that when this parameter is from 0.05 to 0.08. We conclude that the annual inflation rate has a significant impact on the total cost of the original problem. When we design the hazardous waste management system at regions with high economic fluctuations, the annual inflation rate should be carefully evaluated so as to control the cost to design the management system.

**Figure 10:**
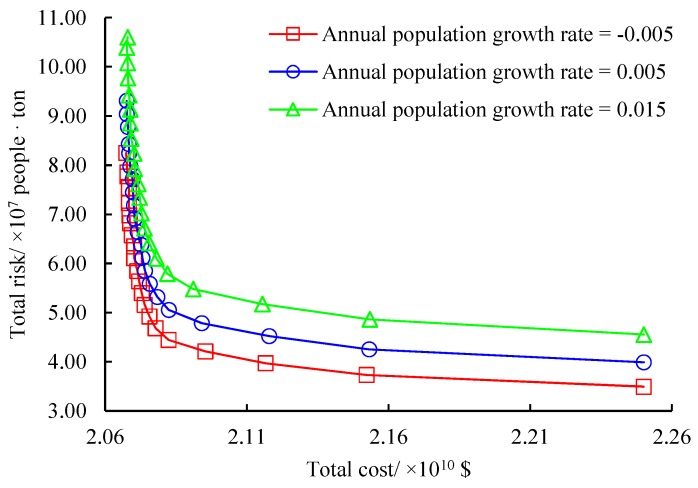
Effects of annual population growth rate.

Then, the impacts of annual population growth rate are analyzed. According to our risk measurement, the population growth rate at each period directly influences the total risk at the period. In total three annual population growth rates are set in accordance with the population development in the test city. They are −0.005, 0.005 and 0.015, indicating a steady decrease, steady increase and rapid increase of population, respectively. The same sensitivity analysis method as for the annual inflation rate is used here and the results are displayed in Figure 10. As shown, the total cost is not impacted by the annual population growth rate due to the defined cost measurement. At the same time, the approximated non-dominated frontiers under the three annual population growth rates have similar variation trends. They are close to each other at the first 15 solutions and then diverge gradually. The average increase of the total risk under the annual population growth rate from 0.005 to 0.015 is 1.24 times of that when this parameter is from −0.005 to 0.005. We can see that the annual population growth rate impacts the total risk of the original problem. To design hazardous waste management systems in regions where the population growth is rapid, the law of population growth in the region should be known clearly if decision-makers want to minimize the total risk.

**Figure 11:**
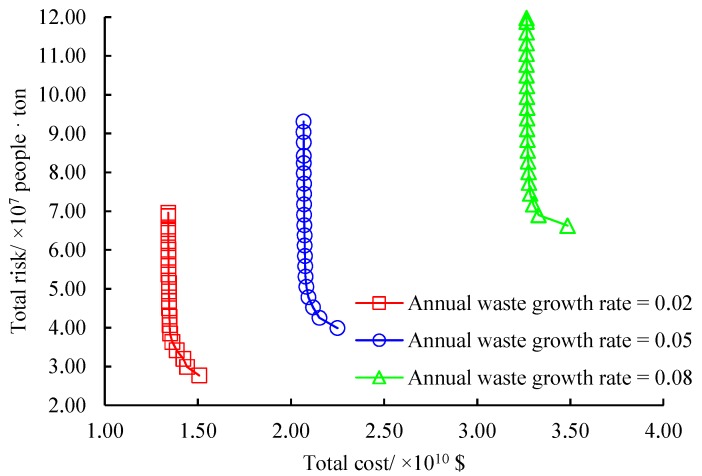
Effects of annual waste growth rate.

We further evaluate the effects of annual waste growth rate. Obviously, both the cost and risk measurements depend on the production of wastes in each period. We consider three annual waste growth rates including 0.02, 0.05 and 0.08 with respect to a slow increase, steady increase and rapid increase of wastes in the test city, respectively. Sensitivity analysis results are provided in Figure 11. We can know from this Figure that the annual waste growth rate influences both the total cost and total risk of the original problem significantly. The three approximated non-dominated frontiers holding similar structures will climb along the diagonal line of the first quadrant with the increase of the annual waste growth rate. The average increase of the total cost when the annual waste growth rate is from 0.05 to 0.08 is 1.65 times of that when the parameter is from 0.02 to 0.05. The total risk has almost the same variation trend as the total cost under the three annual waste growth rates. Thus we indicate that the annual waste growth rate determining the production of wastes is a quite important parameter in our problem. They should be analyzed and predicted reasonably before the long-term multi-period design of regional hazardous waste management systems.

**Figure 12:**
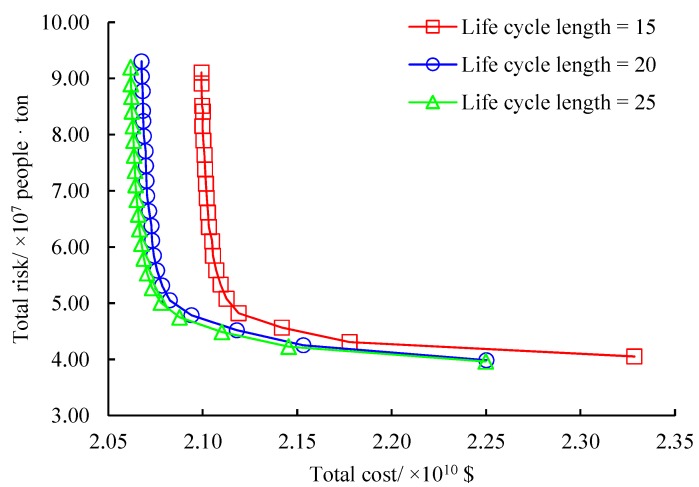
Effects of life cycle length.

Finally, we explore the effects of the life cycle length of disposal centers. In our problem, the life cycle capacity is introduced to reflect the exhaustible resources at disposal centers. We define that the life cycle capacity of a disposal center = annual capacity of the center × life cycle length. Obviously, the life cycle capacity of disposal centers is the right-hand-side value of constraints and will affect both the total cost and total risk of the original problem. Three life cycle lengths of 15, 20 and 25 are considered meaning that each disposal center is built to operate at most 15, 20 and 25 years. Figure 12 gives the sensitivity analysis results for the life cycle length. As indicated in the Figure, the approximated non-dominated frontiers under the three life cycle lengths have similar structures and variation trends. Both the total cost and total risk will be reduced with the increase of the life cycle length. It is interesting that the total cost is more sensitive to the life cycle length than the total risk. The average decrease of the total cost when the life cycle length is from 20 to 25 is just 0.17 times of that when the parameter is from 15 to 20, implying that a life cycle length of 20 years for disposal centers is suitable in the test case. It can be concluded that big disposal centers cannot be guaranteed to reduce the total cost and total risk but they will require large investment and operation cost and might incur much environmental risk. The life cycle capacity of disposal centers should be carefully designed based on the evaluation on the total cost and total risk in the considered regions.

Note that in each of the above four sensitivity analysis, for simplicity, the concerned factor is assumed to have the same variation trend at all districts. However, in practice the variation trend of the factors at districts might be different. Thus, we should carefully predict the factors (e.g., annual inflation rate, annual population growth rate and annual waste growth rate) at each district rather than in the whole region so as to better reflect the unbalance development of districts and to better reveal the impacts of these factors on the design of regional hazardous waste management systems.

## 6. Conclusions

This paper proposes a multi-objective optimization approach for the multi-period network design in regional hazardous waste management systems. We firstly formulate the problem into a bi-objective mixed integer linear programming model to determine the location of waste facilities and allocation/transportation of wastes/residues in each period and minimize the total cost and total risk in the location and transportation procedures of the whole planing horizon. The model considers simultaneously the closing of existing waste facilities, the opening of new waste facilities and the life cycle capacity of disposal centers. According to the characteristics of the problem and the model, an augmented ε-constraint algorithm is developed to find representative non-dominated solutions and balance efficiently the total cost and total risk objectives. Computational results of a practical-sized realistic case show that our algorithm can obtain obviously different and uniformly distributed non-dominated solutions within reasonable time. The effects of the location of waste facilities on the total cost and total risk are bigger than those of the transportation of wastes/residues. Meanwhile, the preference of decision-makers has a significant impact on the multi-period network design strategy for regional hazardous waste management. Besides, compared with the single period optimization, the multi-period network design optimization is necessary and effective to reduce the total cost and total risk in the regional hazardous waste management systems.

Our paper can be extended in several directions. The next work will be dedicated to extend the proposed modeling techniques to describe more network design situations, such as the dynamic opening and closing of waste facilities and the dynamic capacity expansion and extraction of waste facilities, such that the generality of the developed multi-period network design approaches can be improved to better assist the hazardous waste management. Besides, it is worthwhile to introduce more criteria such as the risk equality into the objectives and evaluate the applicability of the augmented ε-constraint algorithm to the multi-objective network design problem. Moreover, our problem contains many coefficients (e.g., costs, risks, rates and capacities) which are not easy to predict precisely. The uncertainty of these coefficients will inevitably impact the design of the hazardous waste management system. It is valuable to investigate the multi-period hazardous waste network design under uncertain coefficients such as uncertain waste production in the future studies.

## Figures and Tables

**Table 1:** Candidate recycling centers.

Recycling Node	Recycling Rate	Fixed Opening Cost ($\times 10^{4}$ $)	Fixed Closing Cost ($\times 10^{4}$ $)	Fixed Operating Cost ($\times 10^{4}$ $/yr)	Variable Process Cost ($/t)	Lower Workload (t/yr)	Upper Capacity (t/yr)	Risk Consequence ($\times 10^{4}$ People)	Risk Probability ($\times 10^{-6}$)
2–15	0.9	3000	150	200	300	5000	10,000	[0.79,2.74]	100

**Table 2:** Candidate treatment centers.

Treatment	Treatment	Residue	Residue	Fixed	Fixed	Fixed	Variable	Lower	Upper	Risk	Risk
Node	Technology	Generation	Recyclable	Opening	Closing	Operating	Process	Workload	Capacity	Consequence	Probability
		Rate	Rate	Cost	Cost	Cost	Cost				
				($\times 10^{4}$ $)	($\times 10^{4}$ $)	($\times 10^{4}$ $/yr)	($/t)	(t/yr)	(t/yr)	($\times 10^{4}$ People)	($\times 10^{-6}$)
2–15	Solidification	1.3	0.2	15,000	750	1000	600	5000	25,000	[0.79,2.74]	400
2–15	Incineration	0.2	0.0	30,000	1500	2000	1200	5000	25,000	[0.79,2.74]	600

**Table 3:** Candidate disposal centers.

Disposal	Disposal	Fixed	Fixed	Fixed	Variable	Lower	Upper	Life	Risk	Risk
Node	Technology	Opening	Closing	Operating	Process	Workload	Capacity	Cycle	Consequence	Probability
		Cost	Cost	Cost	Cost			Capacity		
		($\times 10^{4}$ $)	($\times 10^{4}$ $)	($\times 10^{4}$ $/yr)	($/t)	(t/yr)	(t/yr)	(t)	($\times 10^{4}$ People)	($\times 10^{-6}$)
2–15	Landfill	27,000	13,500	1800	900	5000	30,000	600,000	[0.79,2.74]	200

**Table 4:** Computational results of non-dominated solutions.

#	Total Cost	Total Risk	Computation Time
	($)	(People · ton)	(s)
1	20,676,026,329.8	93,053,886.4	6840.1
2	20,676,290,993.6	90,401,881.3	6480.2
3	20,680,992,688.7	87,742,662.8	6120.5
4	20,683,748,372.7	84,278,640.5	5760.2
5	20,684,303,588.8	82,424,225.8	5400.5
6	20,688,579,661.1	79,746,973.4	5040.1
7	20,697,031,567.1	77,105,788.8	4680.1
8	20,697,984,919.7	74,446,570.2	4321.1
9	20,701,497,158.4	71,787,351.7	3960.1
10	20,704,825,265.3	69,097,504.5	3600.1
11	20,712,097,631.4	66,441,114.6	3240.2
12	20,727,792,658.3	63,788,573.7	2880.1
13	20,731,113,889.2	61,150,477.7	2520.1
14	20,741,294,484.5	58,491,259.2	2160.9
15	20,757,661,992.1	55,832,040.7	1800.1
16	20,784,403,178.3	53,172,822.1	1440.1
17	20,825,111,380.6	50,513,603.6	1080.1
18	20,940,839,801.3	47,854,385.1	720.3
19	21,179,278,507.8	45,195,166.6	360.1
20	21,531,865,418.3	42,535,948.1	57.8
21	22,501,278,056.4	39,876,729.6	1.4
Average			3260.2

**Table 5:** Representative non-dominated solutions (multi-period optimization).

#	Period	Recycling	Treatment	Treatment	Disposal	Total	Total
		Centers	Centers	Centers	Centers	Cost	Risk
			(Tech 1)	(Tech 2)		($)	(People · ton)
1	1	2,7	2,7	2,4,7	2,8	4,032,467,155.4	13,417,995.2
	2	2,7	2,7	2,4,7,8	2,7,8	2,781,239,596.1	14,951,583.8
	3	2,4,7	2,4,7	2,4,5,7,8	2,4,7,8	3,557,848,264.7	18,733,069.8
	4	2,4,5,7	2,4,5,7	2,3,4,5,7	2,4,5,7,8	4,023,538,292.0	22,039,760.8
				8			
	5	2,4,5,7,8	2,4,5,7,8	2,3,4,5,7	2,4,5,7,8	6,280,933,021.5	23,911,476.8
				8,11	11		
					Sum	20,676,026,329.8	93,053,886.4
2	1	2,9	2,9	2,7,13	2,9	4,034,422,517.5	9,433,350.4
	2	2,9	2,9	2,3,7,13	2,9,13	2,788,936,065.9	10,428,807.1
	3	2,9,13	2,9,13	2,3,7,13,15	2,9,13,15	3,569,417,866.7	12,091,936.4
	4	2,3,9,13	2,3,9,13	2,3,7,8,13	2,3,9,13,15	4,032,625,809.9	15,804,776.6
				15			
	5	2,3,7,9,13	2,3,7,9,13	2,3,4,7,8	2,3,7,9,13	6,286,695,371.3	18,682,244.0
				13,15	15		
					Sum	20,712,097,631.4	66,441,114.6
3	1	2,9	2,9,10	2,9,10,14	2,9,10	6,644,062,276.5	5,869,751.5
	2	9,10	9,10	9,10,12,14	9,10,14	2,909,901,720.2	6,312,141.8
	3	9,10,14	9,10,14	9,10,12,13,14	9,10,12,14	3,625,857,963.5	7,738,874.6
	4	9,10,12,14	9,10,12,14	9,10,12,13,14	9,10,12,13,14	4,112,653,780.6	9,193,084.6
				15			
	5	9,10,12,13,14	9,10,12,13,14	9,10,12,13,14	6,9,10,12,13	5,208,802,315.6	10,762,877.0
		15	15	15	14,15		
					Sum	22,501,278,056.4	39,876,729.6

**Table 6:** Representative solutions (single period optimization).

#	Period	Recycling	Treatment	Treatment	Disposal	Total	Total
		Centers	Centers	Centers	Centers	Cost	Risk
			(Tech 1)	(Tech 2)		($)	(People · ton)
1	1	2,7	2,7	2,4,7	2,7	4,029,517,342.5	12,450,811.5
	2	2,7	2,7	2,4,7,8	2,4,7	2,778,059,962.4	15,130,080.3
	3	2,4,7,8	2,4,7,8	2,4,7,8	2,4,7,8	3,292,300,142.7	19,482,053.2
	4	2,4,7,8	2,4,5,7,8	2,4,5,7,8	2,4,5,7,8	3,998,289,177.1	22,688,820.5
	5	2,4,7,8	2,4,5,7,8	2,3,4,5,7,8,	2,3,4,5,7,	7,578,642,683.3	23,195,257.7
				11,13	8,11		
					Sum	21,676,809,307.9	92,947,023.1
2	1	2,15	2,15	2,13,15	2,15	4,034,817,674.7	9,777,504.4
	2	2,15	2,15	2,9,13,15	2,9,15	2,789,513,985.0	10,198,248.5
	3	2,9,13,15	2,9,13,15	2,9,13,15	2,9,13,15	3,311,216,166.8	11,179,811.9
	4	2,9,13,15	2,9,12,13,15	2,9,12,13,15	2,9,12,13,15	4,622,778,191.0	12,391,553.1
	5	2,9,12,13,15	2,9,12,13,15	2,6,7,9,12,	6,7,9,12,13,	7,116,808,513.8	13,718,022.2
				13,15	14,15		
					Sum	21,875,134,531.4	57,265,140.2
3	1	2,9,10	2,9,10	2,9,10	2,9,10,14	6,262,803,336.0	6,583,088.2
	2	2,9,10	2,9,10,14	2,9,10,14	2,9,10,14	2,523,499,826.8	7,487,661.2
	3	2,9,10,14	2,9,10,12,14	2,9,10,12,14	2,9,10,12,14	3,682,431,946.3	8,675,022.0
	4	2,9,10,12,14	2,9,10,12,13,	2,9,10,12,13,	2,9,10,12,13,	4,167,151,377.3	9,748,280.9
			14	14	14		
	5	2,9,10,12,13,	2,9,10,12,13,	2,9,10,12,13,	2,6,7,9,10,11,	8,015,546,047.8	11,722,559.3
		14	14,15	14,15	12,13,14,15		
					Sum	24,651,432,534.2	44,216,611.5
